# Ligustilide‐loaded liposome ameliorates mitochondrial impairments and improves cognitive function via the PKA/AKAP1 signaling pathway in a mouse model of Alzheimer's disease

**DOI:** 10.1111/cns.14460

**Published:** 2023-09-17

**Authors:** Qi Zhang, Xiangxiang Zhang, Bing Yang, Yan Li, Xue‐Heng Sun, Xiang Li, Ping Sui, Yi‐Bin Wang, Shu‐Yu Tian, Chun‐Yan Wang

**Affiliations:** ^1^ Key Laboratory of Major Chronic Diseases of Nervous System of Liaoning Province Health Sciences Institute of China Medical University Shenyang China; ^2^ Key Laboratory of Medical Cell Biology of Ministry of Education Health Sciences Institute of China Medical University Shenyang China; ^3^ Translational Medicine Laboratory, Basic College of Medicine Jilin Medical University Jilin China

**Keywords:** Alzheimer's disease, Ligustilide, liposome, mitochondrial fusion and fission, oxidative stress, PKA/AKAP1 signaling

## Abstract

**Background:**

Oxidative stress is an early event in the development of Alzheimer's disease (AD) and maybe a pivotal point of interaction governing AD pathogenesis; oxidative stress contributes to metabolism imbalance, protein misfolding, neuroinflammation and apoptosis. Excess reactive oxygen species (ROS) are a major contributor to oxidative stress. As vital sources of ROS, mitochondria are also the primary targets of ROS attack. Seeking effective avenues to reduce oxidative stress has attracted increasing attention for AD intervention.

**Methods:**

We developed liposome‐packaged Ligustilide (LIG) and investigated its effects on mitochondrial function and AD‐like pathology in the APPswe/PS1dE9 (APP/PS1) mouse model of AD, and analyzed possible mechanisms.

**Results:**

We observed that LIG‐loaded liposome (LIG‐LPs) treatment reduced oxidative stress and β‐amyloid (Aβ) deposition and mitigated cognitive impairment in APP/PS1 mice. LIG management alleviated the destruction of the inner structure in the hippocampal mitochondria and ameliorated the imbalance between mitochondrial fission and fusion in the APP/PS1 mouse brain. We showed that the decline in cAMP‐dependent protein kinase A (PKA) and A‐kinase anchor protein 1 for PKA (AKAP1) was associated with oxidative stress and AD‐like pathology. We confirmed that LIG‐mediated antioxidant properties and neuroprotection were involved in upregulating the PKA/AKAP1 signaling in APPswe cells in vitro.

**Conclusion:**

Liposome packaging for LIG is relatively biosafe and can overcome the instability of LIG. LIG alleviates mitochondrial dysfunctions and cognitive impairment via the PKA/AKAP1 signaling pathway. Our results provide experimental evidence that LIG‐LPs may be a promising agent for AD therapy.

## Graphical abstract

Ligustilide‐mediated interactions of protein kinase A (PKA) and AKAP1 ameliorate the imbalance between mitochondrial fission and fusion in the brains of AD mouse model, alleviate oxidative stress injury and inhibit apoptosis. The increases in ATP triggered by Ligustilide treatment activate PKA and enhance the transcription of AKAP1, facilitating the upregulation of PKA/AKAP1 signaling.

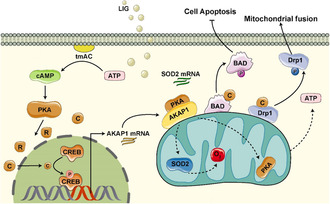

## 1 INTRODUCTION

Alzheimer's disease (AD) is a chronic, progressive, neurodegenerative disease with memory and cognitive deficits as the main clinical manifestation. AD is the most common cause of dementia in aged people, accounting for 60%–80% of dementia patients. Oxidative stress and metabolism imbalance play important roles in synapse loss and neuronal apoptosis during the development of AD.^1^ It is rational to propose that oxidative stress is an early event in AD etiology.^2^ The hallmarks of oxidation, such as lipid peroxidation,^3^ the hydroxyl radical adducts of DNA, DNA oxidation products,^4^ carbonylated proteins^5^ and advanced glycation end‐products^6^ were disclosed in the postmortem brain of patients with mild cognitive impairment.^7^ When redox imbalance occurs, excess reactive oxygen species (ROS) are major contributors to oxidative stress. A series of studies showed that the β‐amyloid (Aβ) peptide,^8^, ^9^ especially Aβ oligomers,^10^ the core component of senile plaques in the brains of patients with familial AD, could directly induce increases in ROS. The interactions of Aβ with metal ions are deleterious to the surrounding biomolecules^11^ except for producing excess ROS. ROS can prime microglia, which release inflammatory cytokines and chemokines, causing chronic inflammatory reactions.^12^, ^13^ Accordingly, oxidative stress facilitates the hydrolysis of amyloid precursor protein to amyloidogenesis.^14^ Increased oxidative stress during AD could activate the inflammatory signals.^15^, ^16^ Importantly, the vital source of ROS is mitochondria. Mitochondrial dysfunction has been reported in AD patients, such as the loss of mitochondrial integrity,^17^, ^18^ decline in complex I and IV activity, impairment of electron transport chain enzymes^19^, ^20^ and decreases in mitochondrial movement, inducing ROS accumulation and promoting oxidative stress. Accordingly, excess ROS cause a decline in the mitochondrial membrane potential and reduce mitochondrial dynamics, leading to ATP synthesis disorder. Under the above conditions, the mitochondrial membrane is vulnerable to oxidative stress. With the increase in protein and lipid peroxidation, the permeability of the mitochondrial membrane is destroyed, causing a further decrease in electron transfer activity, constituting a vicious circle. Oxidative stress may play a pivotal role in AD pathogenesis.^1^ For AD intervention, approaches targeting the reversal of oxidative stress and the restoration of mitochondrial function are now emerging and seem to be promising.

As the main energy source of the brain, ATP participates in biological processes through enzymatic reactions. ATP is catalyzed by activated adenylate cyclase and then condenses to form cyclic adenosine monophosphate (cAMP). As a secondary messenger in cells, cAMP regulates cell functions such as gene transcription, cell migration, cell death and cell metabolism.^21^ The effects of cAMP are mediated by cAMP‐dependent protein kinase A (PKA) and guanylate exchange factor, which are directly activated by cAMP. cAMP can phosphorylate the target protein by activating PKA, regulating the cell response. PKA, as the main effector downstream of cAMP, is a serine and threonine kinase, which consists of two regulatory (R) subunits and two catalytic (C) subunits. When combined with cAMP, the conformation of PKA is changed. The C subunit of PKA (PKA C‐α) exposed can phosphorylate downstream substrates. Importantly, A‐kinase anchor proteins (AKAPs) are essential for anchoring PKA to a specific subcellular structure, ensuring specificity in signal transduction.^22^, ^23^ Mitochondria AKAP (AKAP1, also named dual‐specificity anchoring protein 1) can combine with subunit R of PKA, anchoring PKA onto the outer membrane of mitochondria (OMM) from the cytoplasm.^24^ PKA on the OMM can enhance the binding between AKAP1 and the MnSOD mRNA 3'UTR, promoting the translocation of MnSOD mRNA to mitochondria. Under this condition, the translation of Mn SOD transcripts in mitochondria may be more effective or may contribute to the mitochondrial input of MnSOD, reducing the level of intracellular oxidative stress.^25^ The interaction of cAMP with AKAP1 is a positive feedback loop. The enhancement of cAMP/PKA signaling induces the expression of AKAP1; accordingly, the expression of AKAP1 stimulates the signal transduction of cAMP.^26^ It has been reported that the activities of PKA were reduced by approximately 20% in temporal cortical tissue and 20%–40% in the cerebellum of patients with AD.^27^ cAMP‐binding PKA was significantly lower in the entorhinal cortex with respect to the stage of neurofibrillary changes and was markedly reduced in the entorhinal cortex and subiculum from the postmortem brain of AD patients staged at Aβ deposition.^28^ In vitro, Aβ management significantly inhibited the activity of PKA.^29^ Activation of PKA and upregulation of its downstream target, the SIR2 ortholog Sirtuin1, could reduce Aβ levels.^30^ Importantly, it was reported that endogenous AKAP1 was significantly reduced in the hippocampus and cortex of AD mouse model of 5×Tg beyond Aβ deposition and tau pathology.^31^ Batista and colleagues reported that liraglutide, a drug primarily used to treat diabetes mellitus type 2 and obesity, could diminish the loss of synapses and reverse cognitive impairment by activating PKA signaling.^32^ An antioxidant, genistein, could protect granulosa cells from oxidative stress by upregulating cAMP‐PKA signaling.^33^ Another antioxidant, resveratrol, could improve synaptic plasticity and neuronal structure and function by activating PKA.^34^ The increases in AKAP1 triggered by transient expression could reduce Aβ‐induced mitochondrial fission, dendrite dystrophy, and apoptosis in primary cortical neurons.^31^ The effects of antioxidants on the PKA‐AKAP1 interaction and their role in the regulation of oxidative stress during AD development remain unspecified.

Ligustilide (3‐butylidene‐4,5‐dihydrophthalide, LIG) is a phenol derivative derived from Chinese herbs such as Angelica sinensis and Ligusticum. LIG alleviated mitochondrial damage in a senescence‐accelerated mouse model.^35^ LIG ameliorated neuroinflammation and was neuroprotective in an ischemia model in vivo and in vitro.^36^, ^37^ Interestingly, Xu and colleagues showed that LIG could repair mitochondrial morphology, reduce Aβ levels and mitigate cognitive impairment in an AD mouse model.^38^ However, to date, there is no comprehensive description of the effects of LIG on AKAP1 signaling and/or PKA activity in the treatment of AD pathology. It is worth noting that the unsaturated phenyl hydrazine structure with active butenyl groups of LIG makes it extremely unstable and easily dehydrogenated, hydrolyzed, oxidized and degraded,^39^ which limits its utilization.

In this study, we developed LIG‐loaded liposome and assessed its effects on PKA/AKAP1 signaling and mitochondrial function in the APPswe/PS1dE9 (APP/PS1) double transgenic mouse model of AD. We confirmed that the downregulation of PKA/AKAP1 signaling is accompanied by mitochondrial dysfunction in the brains of APP/PS1 mice. Moreover, LIG induced the upregulation of PKA/AKAP1, reduced oxidative damage and alleviated AD‐like pathology in the APP/PS1 mouse brain.

## 2 MATERIALS AND METHODS

### 2.1 Preparation and characterization of LIG‐LPs

Lecithin (200 mg) and cholesterol (100 mg) were completely dissolved in 10 mL of absolute ethanol in a round‐bottom flask. Then, 1.5 μL of DL‐α‐tocopherol and 200 μL of Tween 80 were added to the solution. To prepare LIG‐LPs, 54.5 μL of LIG was added to the above mixture. After transfer to a 250 mL eggplant‐shaped flask, the solutions were placed onto a rotary evaporator. The absolute ethanol was removed under vacuum at 50°C. When forming a thin film covering the walls of the flask, the sample was hydrated using prewarmed 40 mL of physiological saline (PBS, 0.01 M) until the entire film was suspended to a white suspension. The obtained suspensions were then sonicated (300 W output, 5‐s interval, 15 min). The LPs or LIG‐loaded LPs were collected by filtration with a 0.22‐μm microporous membrane.

Drug release assays in vitro were conducted as previously described.^40^ One milliliter of LIG‐LPs was placed into a dialysis bag (molecular weight cutoff 1400 Da), which had been pretreated against deionized water at 100°C for 10 min. The dialysis bag with the sample was placed into 50 mL of 0.01 M PBS (containing 1% Tween‐80, pH 7.4) with gentle stirring at 100 rpm (37°C). Aliquots of the supernatant were withdrawn and diluted with nine volumes of methanol at the indicated times (1, 2, 4, 12, 24, 36 and 72 h). The absorbance of the sample was measured at 315 nm by spectrophotometry. The same volume of fresh PBS was added back to the dialysis container at each time point. The results were represented as the cumulative release ratio of LIG that had permeated at the indicated time (assuming 100% permeation at infinite time).

The assays of encapsulation and loading efficiency were performed according to the description^41^ with minor revision. Briefly, after centrifugation at 3000 rpm for 30 min, the LPs‐encapsulated LIG was separated from free LIG. Subsequently, the dissolved LIG in the supernatant was detected by UV spectrophotometry at 315 nm. The encapsulation efficiency (EE) and loading efficiency (LE) were calculated as follows.

$$\mathrm{EE}\;\left(\%\right)=\frac{\text{Total amount of}\;\mathrm{LIG}\;\text{in formulation}-\text{Amount of unbound}\;\mathrm{LIG}}{\text{Total amount of}\;\mathrm{LIG}\;\text{in formulation}\;}\times 100;$$

$$\mathrm{LE}\;\left(\%\right)=\frac{\text{Total amount of}\;\mathrm{LIG}\;\text{in formulation}-\text{Amount of unbound}\;\mathrm{LIG}}{\text{Total amount of in formulation}}\times 100.$$

The particle size and zeta potentials of LIG‐LPs were determined by laser diffraction or Doppler velocimetry at 25°C on a Zetasizer Nano ZS90 instrument (Malvern Instruments Ltd.). All measurements were performed at least three times.

The morphology of the LIG‐LPs was detected with a transmission electron microscope (TEM) (JEM‐2100; Nikon). Briefly, 10‐μL samples were dropped onto copper mesh. After 10 min, 10 μL of 5% phosphotungstic acid solution was added to the sample on the copper mesh. The excess liquid was removed, and the sample was dried in an incubator at 37°C. LIG‐LPs and the LPs without drug loading were observed at an accelerating voltage of 200 kV.

### 2.2 Safety assessment of LIG‐LPs

To assess the toxicity of LIG‐LPs in vivo, male C57BL/6 mice at the age of 4 months (body weight: 22–26 g) were randomly divided into three groups: intraperitoneally injected with saline, LPs or LIG‐LPs (*n* = 8 in each group) once per day for 30 days. The body weight and general health of the mice were monitored. On day 31, the mice were intraperitoneally anesthetized with 60 mg/kg sodium pentobarbital. After transcardial perfusion with prechilled saline (0.9%), the mice were sacrificed by decapitation. The heart, liver, spleen, lung and kidney were collected. After fixation in 4% paraformaldehyde, the tissues were dehydrated with 30% sucrose. Then, serial sections (10 μm thick) were collected using a freezing microtome and stained with hematoxylin and eosin (H&E).

### 2.3 Animals and treatment

Male APP/PS1 (B6C3‐Tg [APPswe, PSEN1dE9] 85Dbo/Mmjax) transgenic mice (RRID: MMRRC_034829‐JAX) aged 4 months were randomly assigned to three groups: one LPs‐treated group and two groups treated with LPs‐encapsulated LIG at doses of 10 and 30 mg/kg. The animals were intraperitoneally injected daily with the indicated treatment for 6 months. The body weights and general behaviors of the mice were monitored. This study was performed in accordance with the recommendations of Laboratory Animals‐Guideline of Welfare and Ethics, the Ethics Committee for Medical Laboratory Animals of China Medical University. The protocol of animal experiments was approved by the Ethics Committee for Medical Laboratory Animals of China Medical University (CMU2020003).

### 2.4 Evaluation of cognition

Nest construction tests were conducted to assess the affiliative social behavior of the mice. During the 7‐day test, the mice were housed individually in cages containing 1‐cm‐deep corncob bedding and eight pieces of paper (5 cm × 5 cm). The nesting behavior of mice was examined in the morning every day. The nesting scores of each mouse were measured in accordance with a four‐point scoring system.^42^

The novel object recognition (NOR) test was performed to investigate the recognition memory of the mice. The mice underwent 2 days of habituation in an arena (50 cm length × 50 cm width × 40 cm height) twice a day (10 min each time). On the 3rd day, two plastic blocks of the same volume were placed in the arena. The mouse was allowed to explore the blocks for 5 min. When the mouse was subjected to the short‐term memory (STM) test, it was placed back cage, in which one of the blocks was replaced by a plastic cylinder. When long‐term memory (LTM) was evaluated, the interval was 24 h between exploring the arena containing two blocks and moving in the arena with one novel cylinder. The definition of exploratory behavior was as follows: the mouse touched an object with its nose and/or forepaws and/or directed its nose toward the object within a 2‐cm distance. The discrimination index represents the percentage of time taken to explore the novel object relative to the time taken to explore both objects.

The Morris water maze (MWM) test was implemented to assess the spatial learning and memory of the mice. The mice were given 2 days of training to swim in a water maze with a visible platform. Then, the platform was hidden 1 cm below the water surface. During the following 5 days of the navigation test, the mice were placed inside the tank and allowed to swim freely. The time that the mice spent finding the hidden platform was recorded as the escape latency. In the probe trial on the 8th day, the platform was removed. The times that the mice passed across the region in which the platform had previously been placed were compared.^43^

### 2.5 Immunostaining

After the behavioral tests, the mice were deeply anesthetized and transcardially perfused using precooled 0.9% saline. The brains were collected and dissected into two blocks by means of a single sagittal cut. The right hemisphere was fixed for morphological analyses. The left hemisphere was prepared for biochemical assays. After fixation and dehydration, 10‐μm frozen sections from brain tissue were prepared with a freezing microtome. To eliminate endogenous peroxidase activity, the sections were treated using 3% hydrogen peroxide. After rinsing, the brain slices were blocked with 5% normal donkey serum (30 min, room temperature). According to the protocol for immunohistochemistry (IHC) or double immunofluorescence (IF), the sections were treated with a primary antibody or a mixture of two kinds of primary antibodies for 16–18 h in a humidified chamber (4°C). The sections were then thoroughly rinsed. The brain slices subjected to IHC staining were treated with an appropriate biotinylated secondary antibody (2 h, room temperature), followed by incubation with streptavidin peroxidase (30 min, room temperature). After several rinses, the sections were treated with chromogenic solution (0.025% 3,3‐diaminobenzidine, 0.0033% H_2_O_2_, 0.1 M Tris‐HCL, pH 7.4). After dehydration and clearing, the slices were mounted with neutral balsam and examined with an optical microscope (Olympus, Model DP72).

For double IF staining, the sections were treated with a mixture of secondary antibodies (appropriate fluorescence‐conjugated) for 2 h (room temperature, away from light). 4′,6‐diamidino‐2‐phenylindole (DAPI) was used to stain nuclear chromatin. The sections were treated using an anti‐fading mounting medium and prepared to be scanned by a confocal laser scanning microscope (Nikon 1).

### 2.6 Golgi staining

Golgi staining was conducted with an FD Rapid GolgiStain™ Kit (FD Neurotechnologies, Inc.) according to the manufacturer's instructions. The right hemisphere of the mouse brain was collected and immersed in an impregnation solution (containing mercuric chloride, potassium dichromate and potassium chromate) for 24 h. Then, the tissue block was incubated in fresh impregnation solution for 2 weeks protected from light. After that, the brain hemisphere was immersed in Solution C at 4°C for 24 h. During the following day, the tissue was incubated with fresh Solution C for another 24 h in the dark. After impregnation, the block was removed from the solution. Excess liquid on the tissue was removed with filter paper. The tissue was quickly frozen in liquid nitrogen. Frozen slices with a thickness of 50 μm were prepared using a flat‐push microtome. After being washed twice with precooled double distilled water, the sections were treated with a dying solution for 10 min. After washing with double distilled water, the slices were dehydrated, cleared and covered by neutral balsam. Sholl analysis^44^ was used to evaluate the morphological structure of dendrites. The number of dendritic intersections corresponding to the images generated from cell body‐centered concentric circles (radial distances: 10–250 μm with radii 10 μm apart) was calculated. The density of spines in the hippocampal CA1, CA3 and dentate gyrus (DG) was also calculated according to the number of spines per unit area.

### 2.7 Assessment of mitochondrial morphology by TEM

One cubic millimeter of hippocampal tissue from the same regions in the mice was fixed in 2.5% glutaraldehyde solution overnight. After rinsing with 0.01 M PBS, the tissues were fixed with osmium acid for 1 h. The tissues were washed with PBS and gradient dehydrated with ethanol and acetone. After embedding with epoxy resin 618, the tissue was cut into 50‐nm‐thick sections. The samples were stained with lead citrate and uranyl acetate and were photographed by TEM.

### 2.8 Evaluation of oxidative redox

To analyze the status of lipid peroxidation, malondialdehyde (MDA) contents in the mice brains were examined by the method of colorimetry with an MDA Detection Kit (Nanjing Jiancheng Bioengineering Institute) according to the manufacturer's instructions. MDA can be precipitated by thiobarbituric acid. The products were recorded at 532 nm using a photometer.

The levels of carbonyl proteins in the mice brains were assessed as previously described.^45^ Briefly, the total protein of each sample was treated with 2,4‐dinitrophenylhydrazine at a concentration of 1% in hydrochloric acid (2 M) for 1 h (room temperature). The samples were collected and resuspended in trichloroacetic acid (20%), followed by the extraction with ethanol/ethyl acetate (1:1). The supernatants were removed. The pellets were lysed with lysis buffer (pH 7.4) containing 8 M guanidine hydrochloride, 13 mM EDTA, and 133 mM Tris. The absorbance was read at 365 nm.

To assess the ratio of reduced glutathione (GSH) to oxidative glutathione (GSSG), the contents of GSH and GSSG were examined according to previously described.^46^ Briefly, the mice brains were treated with precooled PBS (m/v, 1:10) containing a protease inhibitor cocktail. The supernatants were collected by centrifugation. The levels of GSH and GSSG were measured with colorimetric assay kits (Beyotime Institute of Biotechnology). The contents of total GSH (T‐GSH) were determined by the method of 5,5‐dithiobis (2‐nitrobenzoic acid)‐GSSG recycling assay. The quantification of GSSG is based on the principle: reduced GSH reacts with 5,5′‐dithiobis (2‐nitrobenzoic acid), producing 5‐thio‐2‐nitrobenzoic acid. The optical density was read at 412 nm. The contents of GSH were obtained by subtracting the GSSG contents from the T‐GSH.

### 2.9 Flow cytometry (FCM) assays

Cells of the cerebral cortex were collected. Briefly, cortical tissues were minced with scissors. The fragments were lysed with 20 mg/mL papain for 1 h at 37°C. After centrifugation at 400× *g* for 10 min, the precipitates were resuspended in 1 mg/mL deoxyribonuclease I for 1 h at 37°C. After filtered through a 70‐μm mesh screen, the specimens were rinsed with PBS and collected by centrifugation. The samples were treated with a separating solution (20% Percoll in Hanks' balanced salt buffer). The cells were then collected and washed with prechilled buffer (10 mM HEPES, 140 mM NaCl, and 2.5 mM CaCl_2_, pH 7.4).

To assess ROS levels, cells at a concentration of 2 × 10^6^/mL were treated with 1 μM CM‐H_2_DCFDA (5‐(and‐6)‐chloromethyl‐2′,7′‐dichlorodihydrofluorescein diacetate, acetyl ester) (Invitrogen™) at 37°C for 30 min away from light. After washing, the samples were treated with CM‐H_2_DCFDA solution for another time (15 min, 37°C). After lysed with lysis buffer, the samples were collected by centrifugation and were diluted in double distilled water. ROS production was measured by FCM assays with a VersaFluor Fluorometer System (Bio‐Rad Laboratories) at 492 nm excitation and 527 nm emission.

For determining the degree of mitochondrial membrane polarization, cells were incubated with 2 μM JC‐1 (5′,6,6′‐tetrachloro‐1,1′,3,3′‐tetraethylbenzimidazolylcarbocyanine iodide) solution (MitoProbe™) at 37°C for 30 min in the dark. Cells were washed once with prewarmed PBS. The samples were resuspended in PBS and examined by using the emission filters for Alexa Fluor 488 and R‐phycoerythrin.

To evaluate the levels of apoptosis, the cells were fixed in 75% ethanol at 4°C for 1 h. The apoptotic ratio of the cells was counted with an Annexin V‐fluorescein isothiocyanate (FITC)/propidium iodide (PI) apoptosis detection kit. The cells were treated with binding buffer containing 5 μL of Annexin V‐FITC and 5 μL of PI at 4°C for 10 min in the dark. Apoptosis was determined using FITC (FL1 channel) and PI (FL2 channel) fluorescence detectors.

### 2.10 Measurement of ATP levels

The analysis of ATP contents was implemented with an Enhanced ATP Assay Kit (Beyotime Biotechnology, S0027). Briefly, tissues from the cerebral cortex were treated with lysis solution (m/v, 1:5) and homogenized by sonication (20 W output, 3 s interval, 2 min) on ice. After lysis for 2 h (4°C) with gentle horizontal rotation, the samples were centrifuged at 12,000× *g* for 30 min. Seventy microliters of supernatant was boiled for 2 min and prepared for ATP analysis. The remaining samples were used to analyze the protein concentration. One hundred microliters of detection solution were added to a 96‐well plate. After 5 min, 70 μL of specimen or standard was added to the well and quickly blended. Subsequently, the samples were read by a spectrophotometer (BioTek CYTATION 5) under luminance mode. The levels of ATP were calculated in accordance with the calibration curve of the ATP standard. The value was normalized to the protein concentration.

### 2.11 Cell culture and treatment

N2a cells or N2a cells stably transfected with human Swedish mutant APP (APPswe) were cultured in DMEM/F12 medium plus 10% FBS, 100 U/mL penicillin and 100 μg/mL streptomycin (fully humidified, 5% CO_2_, 37°C). APPswe cells were treated with G418 (200 μg/mL). When the cells were ~80% confluent, the medium was changed to serum‐free DMEM/F12 medium. The cells were then treated with the indicated intervention, H_2_O_2_, LIG or H89 (PKA inhibitor).

### 2.12 Western blot assays

Proteins were extracted from tissue homogenates or cells with radioimmunoprecipitation assay buffer (RIPA, pH 8.0) containing a protease inhibitor cocktail. The concentrations of proteins were determined with a Bicinchoninic Acid Assay kit (Beyotime Biotechnology, P0012). Sodium dodecyl sulfate–polyacrylamide gel electrophoresis was used to separate the proteins. The proteins were transferred onto polyvinylidene fluoride membranes (Millipore). The proteins on the PVDF membranes were probed by using the primary antibodies presented in Table S1. After incubation with appropriate secondary antibodies, the immunological complex bands were observed using an enhanced chemiluminescence kit (Pierce) and quantified with Quantity One software. Blots were repeated at least three times for every condition.

### 2.13 Statistical analysis

All data are presented as the mean ± standard error of the mean (SEM), and tests for the homogeneity of variance were calculated. The data exhibited a normal distribution. Statistically significant differences between groups were determined with Student's *t* test, repeated‐measures analysis of variance (ANOVA), one‐way ANOVA, two‐way ANOVA or multivariate ANOVA followed by post hoc Fisher's least significant difference (LSD) tests as appropriate. *p* < 0.05 was considered significant.

## 3 RESULTS

### 3.1 The size of the particles was small, and LIG‐LPs were uniformly dispersed and relatively safe

**FIGURE 1:**
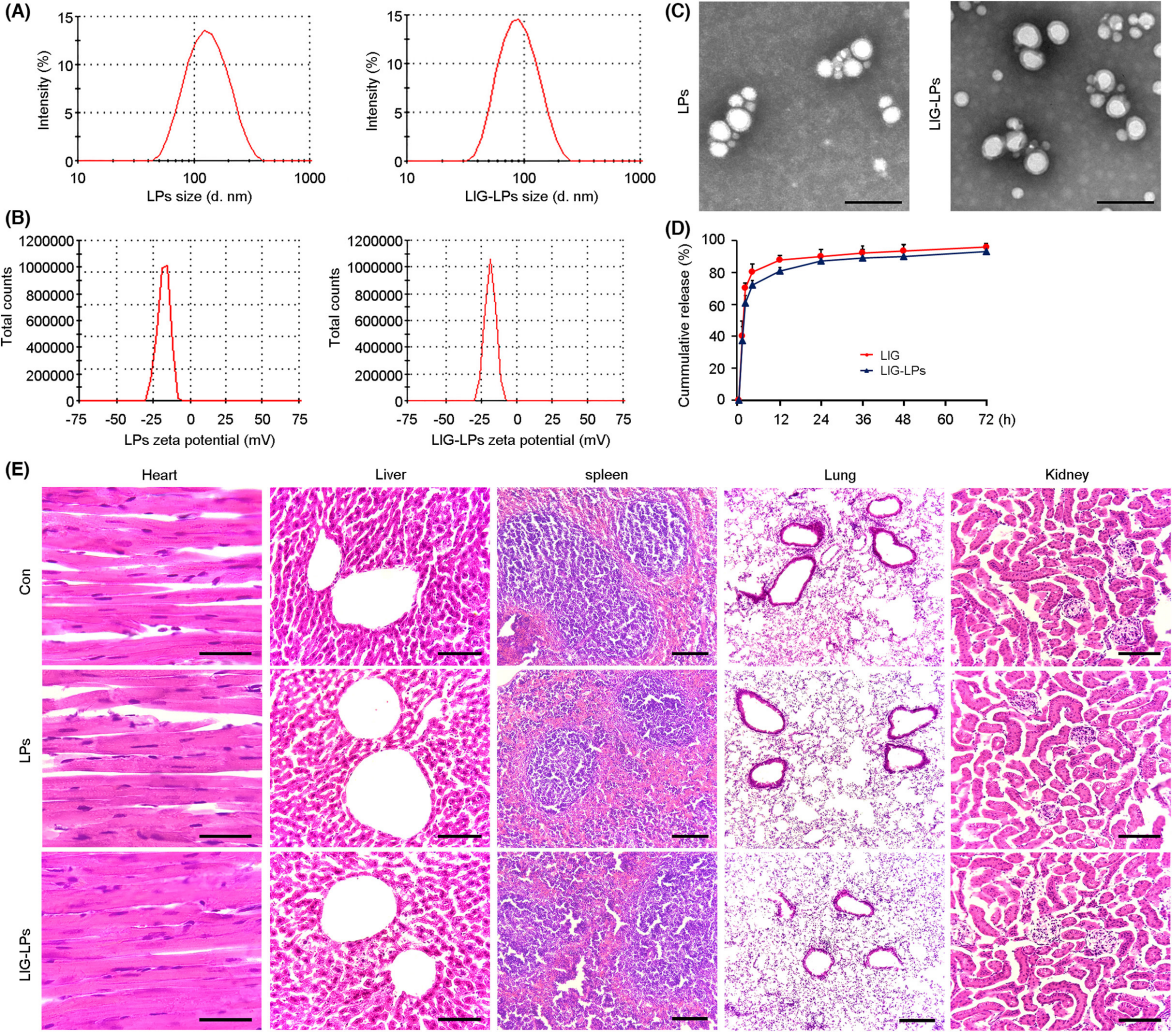
Characterization of Ligustilide‐liposome. (A) Size distribution of liposome (LPs) or ligustilide‐loaded liposome (LIG‐LPs) was determined by dynamic light scattering assays. (B) Zeta potential of LPs or LIG‐LPs showing the charge conditions of the nanoparticles. (C) Representative images exhibited the morphology of LPs or LIG‐LPs by using a transmission electron microscope (TEM). Scale bar = 200 nm. (D) LIG release from LIG‐LPs in PBS in vitro. (E) Hematoxylin and eosin (H&E) staining showing the structure of heart, liver, spleen, lung and kidney from the C57BL/6 mice under LPs or LIG‐LPs administration by intraperitoneal injection (IP) for 30 days. The mice treated with 0.9% sodium chloride by PI were used as the controls (Con). No significant cytotoxic effects were observed. Scale bars: Heart, 20 μm; Liver, 120 μm; Spleen, 200 μm; Lung 200 μm; Kidney, 120 μm.

Considering the unstable nature of LIG, we formulated LIG‐LPs by thin‐film rehydration methods. Liposome as a drug delivery system possess advantages, such as biocompatibility, good solubilization, high loading capacity, increased half‐life and enhanced stability of incorporating medicine and protecting the drugs against degradation in the physiological environment.^47^ The ratio of lecithin to cholesterol (v/v, 2:1) was intended to prevent the structure and property of drugs from being destroyed.^48^ The mean diameters/particle size, zeta potential and polydispersity indexes (PDIs) of the LPs and LIG‐LPs were assayed by laser diffraction or Doppler velocimetry (Figure 1A,B; Table S2). TEM analysis showed that the system was uniform (Figure 1C). LPs and membranes were spherical in shape. The surface morphologies of the LPs and membranes are homogeneous, distinct and discrete. The bilayer area of the LIG‐LPs group appeared murky. The release of LIG from the LPs was monitored at pH 7.4 (37°C). In the first 4 h, continuous release of LIG from LIG‐LPs was presented, rather than a burst of release from the formulations. Then, the release of LIG gradually slowed with time (Figure 1D). To investigate the biological safety of LIG‐LPs, the heart, liver, spleen, lung and kidney were collected from mice that had been given LIG‐LPs by intraperitoneal injection for 30 days. The frozen sections of the above tissues were subjected to H&E staining. No significant tissue damage or inflammatory cell infiltration was observed (Figure 1E).

### 3.2 LIG‐LPs treatment improves cognition in the APP/PS1 mice

**FIGURE 2:**
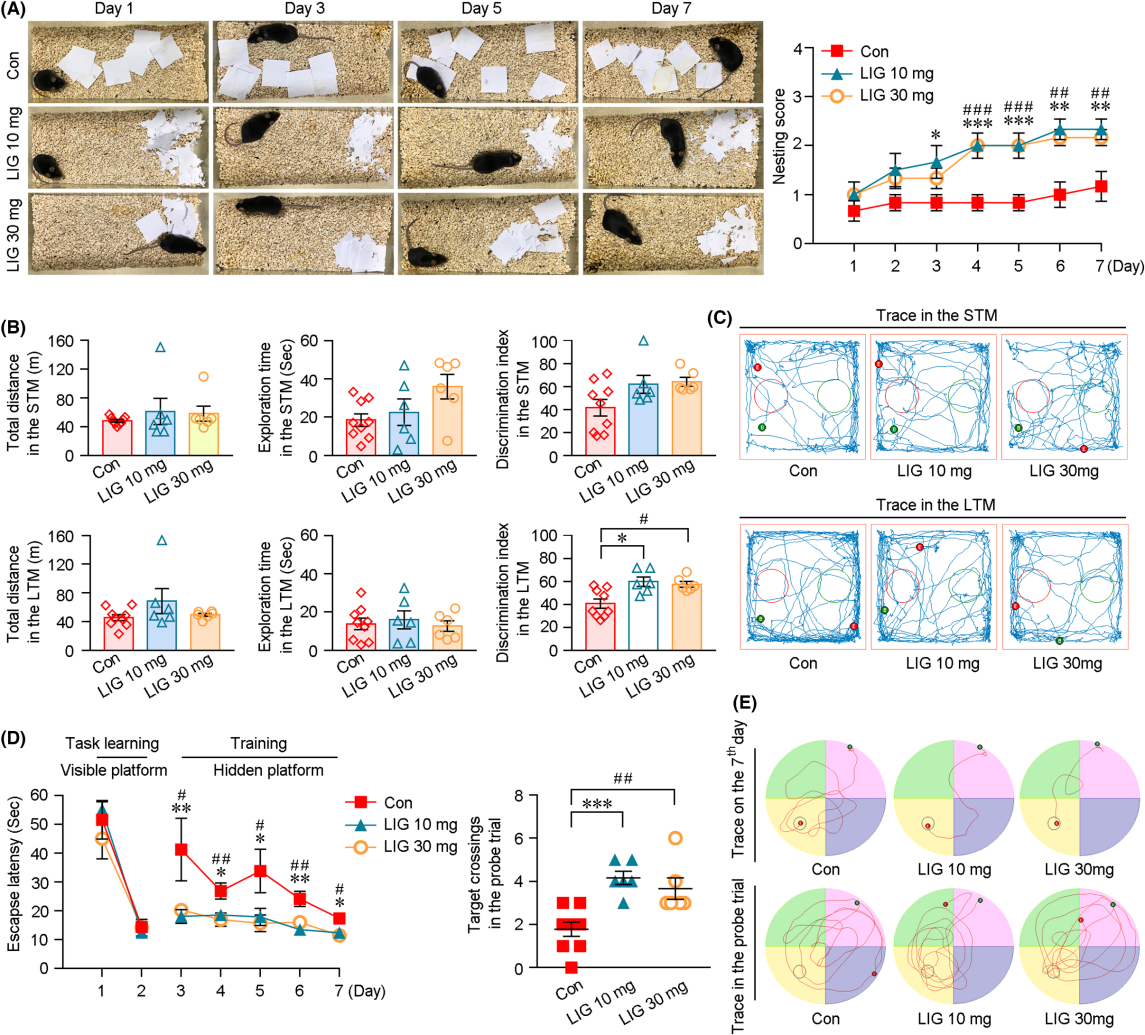
Effects of LIG‐LPs management on the cognitive function in the APP/PS1 mice. LIG‐LPs (at doses of 10 or 30 mg/kg of LIG) were given to male APP/PS1 mice (at the age of 4 months) by IP for 6 months. LPs‐treated APP/PS1 mice were used as the vehicle controls (Con). (A) Representative images exhibited the nesting behavior of the mice. (B) The NOR test was performed to analyze the recognition memory of the mice. The total distance and exploration time were recorded during the STM and LTM tests. (C) Representative images showing the exploration trajectories of the mice. The green dots and red dots, respectively, represented the beginning and the ending locations of the mice. (D) The MWM test was conducted to assess the spatial learning and memory of the mice. The test consisted of 2 days of task learning with a visible platform, 5 days of training with a hidden platform and a probe trial for 1 day without platform. Compared with the controls, the mice under LIG‐LP treatment reached the target more quickly during the training procedure, and crossed the platform area more often than those of the controls in the probe trial. (E) Representative images showing the swim traces of the mice on the 7th and 8th day. All values represent mean ± SEM, *p < 0.05, **p < 0.01 and ***p < 0.001: LIG‐LPs‐treated mice (10 mg/kg) versus Con; ^#^ *p* < 0.05, ^##^ *p* < 0.01 and ^###^ *p* < 0.001: LIG‐LPs‐treated mice (30 mg/kg) versus Con by repeated‐measures ANOVA. *n* = 6–9 in each group.

The effects of LIG‐LPs on the cognition of APP/PS1 mice were investigated by nest construction test, NOR test and MWM test. As shown in Figure 2A, compared with the mice treated with LPs without LIG incorporation (Con), LIG‐LPs‐administrated mice exhibited better nesting behavior. From the third day, the nesting scores of the LIG treatment group were higher than those of the vehicle‐treated group. Quantified assays of NOR test showed that the discrimination index of LIG‐treated mice was increased during the LTM test (Figure 2B,C). The mice in the LIG‐treated group exhibited significant improvement in spatial learning ability during the MWM test (Figure 2D,E). The escape latency of LIG‐treated mice was shortened relative to that of vehicle‐treated group in the hidden platform test. In the probe test, LIG‐treated mice exhibited a significant preference for the region where the platform had been placed compared with the vehicle controls.

### 3.3 LIG‐LPs administration ameliorates AD‐like pathology in the brains of APP/PS1 mice

**FIGURE 3:**
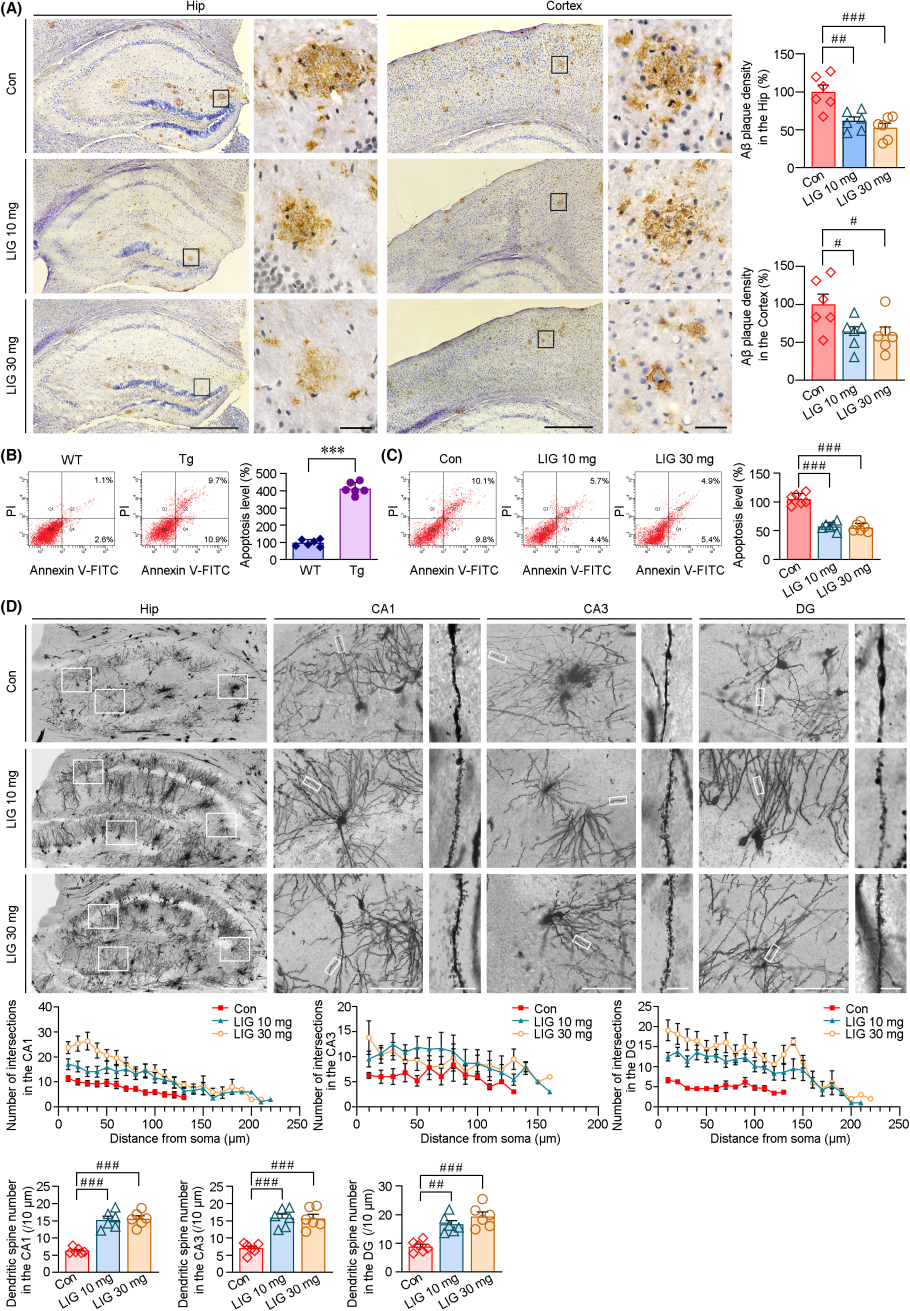
LIG‐LPs administration reduces Aβ‐plaque deposition and provides neuroprotective effects on the neurons of the APP/PS1 mice brains. (A) IHC staining with an anti‐Aβ antibody showed that the Aβ‐plaque accumulations were reduced in the hippocampus (Hip) and cortex under LIG‐LPs treatment, scale bars: 450 μm. The high magnification images in the right panels correspond to the regions indicated in the boxes, scale bars: 50 μm. ^#^ *p* < 0.05 and ^###^ *p* < 0.001 versus vehicle‐treated APP/PS1 mice (Con) by one‐way ANOVA with post hoc Fisher's LSD. (B) FCM assays showed that the apoptosis in the brains of transgenic (Tg) APP/PS1 mice (aged 10 months) was higher than that of age‐matched wild‐type (WT) C57BL/6 mice. ***p < 0.001 versus WT group by the Student's *t* tests. Under LIG‐LPs treatment, the apoptotic ratio was significantly reduced compared with that of vehicle controls in the APP/PS1 mice brains (C). (D) Representative images of Golgi staining showed that LIG treatment alleviated the damage of dendrites in the hippocampus (Hip) of APP/PS1 mice brains. Scale bar = 600 μm. The high‐magnification images correspond to the regions indicated in the boxes. Scale bars were 50 and 10 μm, respectively. The number of dendritic intersections represented the complexity of dendrites. The spine density was calculated to assess the loss of dendritic spines. ^#^ *p* < 0.05, ^##^ *p* < 0.01 and ^###^ *p* < 0.001 versus Con by one‐way ANOVA with post hoc Fisher's LSD (C, D). All values represent mean ± SEM, *n* = 6 in each group.

Considering the pathological characteristics of the AD brain, the effects of LIG‐LPs on Aβ deposition and neuronal damage were assessed. As shown in Figure 3A, IHC staining indicated that Aβ‐positive senile plaques were evident in the hippocampus (Hip) and cerebral cortex of the APP/PS1 mice. The intensity of the immunoreactive product of Aβ in the LIG‐treated mice was reduced compared with that in vehicle‐treated APP/PS1 group. We then analyzed the apoptosis in the cerebral cortex of the mice by FCM. As shown in Figure 3B, the apoptotic cells were significantly increased in the transgenic APP/PS1 mice (Tg) compared with those of age‐matched wild‐type C57BL/6 (WT) mice. Under LIG‐LPs treatment, the number of apoptotic cells was reduced in the cerebral cortex of APP/PS1 mice (Figure 3C). In addition, Golgi staining demonstrated the neuroprotective effects of LIG. As shown in Figure 3D, the damage of dendrites and the loss of spines in the hippocampal neurons were significant in the vehicle‐treated APP/PS1 mice. Under LIG‐LPs treatment, the number of dendritic intersections in the regions of CA1 (at a distance of 10–120 μm from the center of the cell soma), CA3 (10–30 μm) and DG (10–130 μm) were increased compared with those of vehicle controls. LIG‐LPs management also alleviated the loss of spines compared with those of vehicle‐treated controls in the APP/PS1 mice.

### 3.4 LIG‐LPs treatment ameliorates oxidative stress in the APP/PS1 mice brains

**FIGURE 4:**
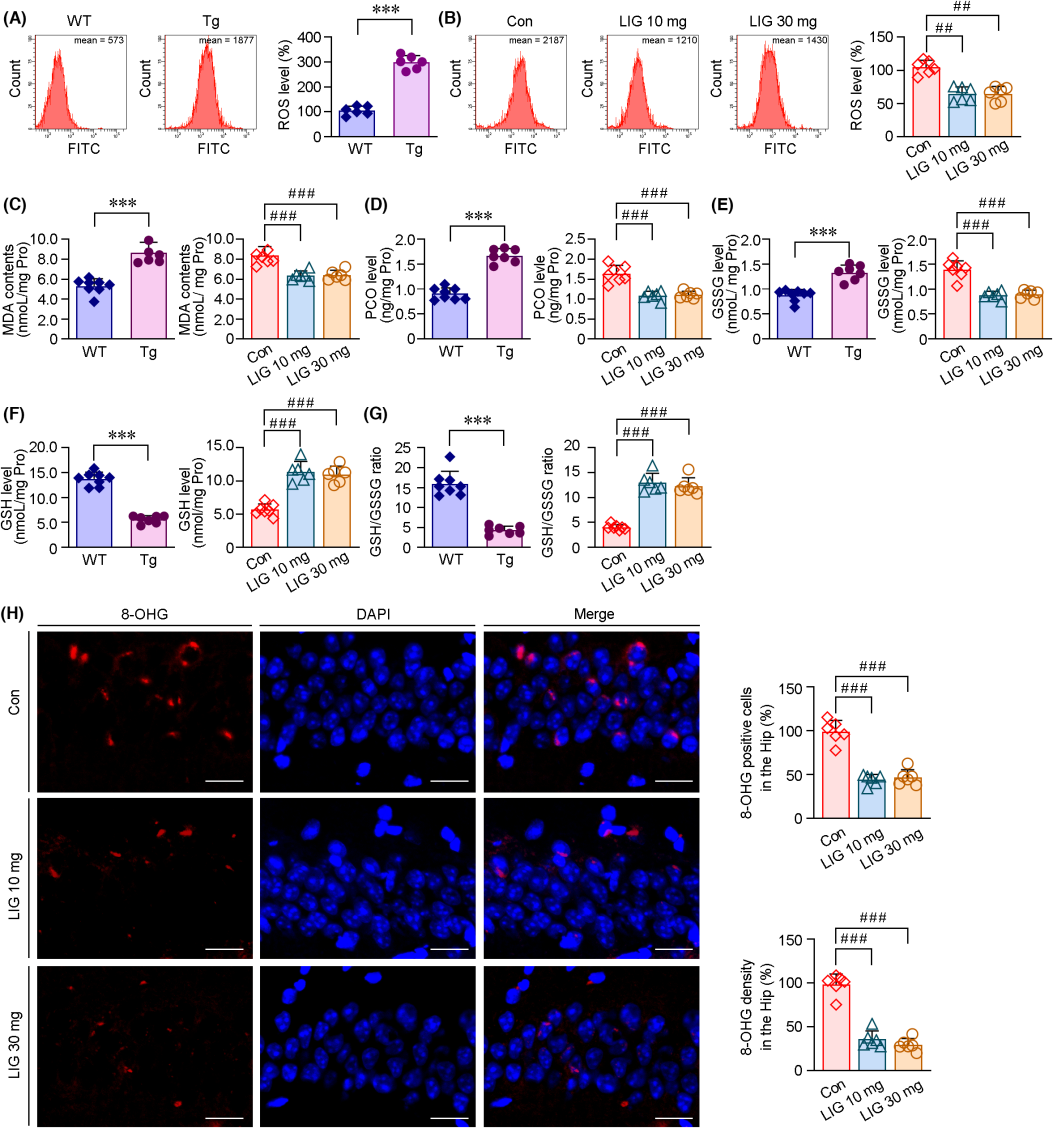
Effects of LIG management on the modulation of redox in the APP/PS1 mice brains. (A) FCM analysis showed that the ROS levels in the transgenic (Tg) APP/PS1 mice brains were markedly higher than that of age‐matched wild‐type (WT) C57BL/6 mice brains. LIG‐LP treatment reduced the ROS contents compared with those of vehicle controls (Con) in the APP/PS1 mice brains (B). (C) The production of lipid peroxidation, MDA, was measured by colorimetric assays. The MDA levels were higher in the Tg mice brains relative to those of age‐matched WT group. LIG‐LPs treatment mitigated the increases of MDA compared with that of vehicle controls in the brains of APP/PS1 mice. (D) The levels of PCO in the brains of Tg and WT mice were compared. LIG‐LP treatment alleviated PCO relative to that of vehicle‐treated group in the APP/PS1 mice brains. (E) LIG management mitigated the increases of GSSG in the APP/PS1 mice brains. The GSH levels (F) and GSH/GSSG ratio (G) were determined. (H) Representative images showing the DNA damage in the hippocampal neurons by IF staining with an antibody against 8‐OHG. Scale bar = 30 μm. The immunoreactive cells were calculated. All values represent mean ± SEM, ***p < 0.001 versus WT group by the Student's *t* tests; ^##^ *p* < 0.01 and ^###^ *p* < 0.001 versus Con by one‐way ANOVA with post hoc Fisher's LSD, *n* = 6–8 in each group.

ROS levels were analyzed in the brains of APP/PS1 mice. As shown in Figure 4A, FCM assays showed that ROS contents in the prefrontal cortex of the APP/PS1 mice brains were higher than those of age‐matched WT controls. ROS levels in the brains of LIG‐LPs‐treated APP/PS1 mice were lower than those of vehicle‐treated APP/PS1 group (Figure 4B). We then investigated the levels of lipid peroxidation and protein carbonyl (PCO). As shown in Figure 4C, MDA levels were significantly increased in the APP/PS1 mice brains compared with those of age‐matched WT group. LIG‐LPs management reduced the MDA levels compared with those of vehicle‐treated APP/PS1 group. The effects of LIG‐LPs treatment on PCO were also observed (Figure 4D). To investigate the redox alterations, the contents of GSH and GSSG and the ratio of GSH to GSSG were assayed. As shown in Figure 4E, GSSG contents were higher in the brains of APP/PS1 mice than those of age‐matched WT controls, whereas GSH levels were lower (Figure 4F). LIG‐LPs management mitigated the decreases in GSH levels (Figure 4F) and GSH/GSSG ratio (Figure 4G) in the APP/PS1 mice brains. DNA damage in the hippocampus was examined with an anti‐8‐hydroxyguanosine (8‐OHG) antibody by IF staining. As shown in Figure 4H, the expressions of 8‐OHG were reduced under LIG‐LPs administration in the APP/PS1 mice brains compared with those of vehicle‐treated APP/PS1 group.

### 3.5 LIG‐LPs management mitigates the mitochondrial damage in the brains of APP/PS1 mice

**FIGURE 5:**
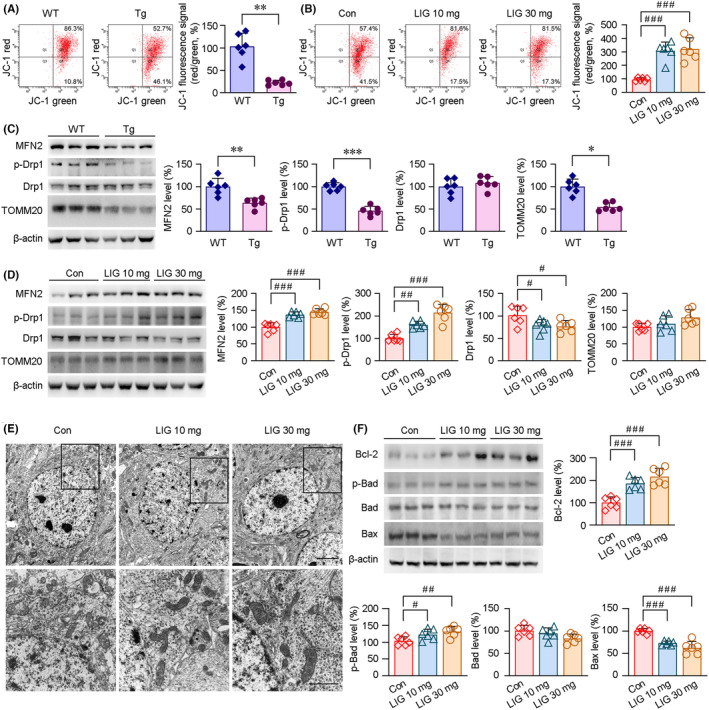
Modulations of LIG treatment on the mitochondrial fusion and fission, and its effects on apoptotic‐related protein in the APP/PS1 mice brains. (A) FCM assays showed that the mitochondrial transmembrane potentials (MMP) were lower in the transgenic (Tg) APP/PS1 mice brains than that of age‐matched wild‐type (WT) C57BL/6 mice brains. LIG management alleviated the decline of MMP in the APP/PS1 mice brains (B). (C) Compared with the WT group, the protein levels of MFN2, p‐Drp1 and TOMM20 were reduced in the APP/PS1 mice brains. LIG administration mitigated the imbalance of mitochondrial fusion and fission (D). (E) TEM analysis showed the ultrastructure of the hippocampal neurons in the APP/PS1 mice brains, scale bars: 2 μm. The high magnification images showed the mitochondria corresponding to the regions indicated in the boxes, scale bars: 1 μm. (F) Western blot assays showing the LIG‐mediated effects on the protein expressions of Bcl‐2, p‐Bad, Bad and Bax. All values represent mean ± SEM, *p < 0.05, **p < 0.01 and ***p < 0.001 versus WT group by Student's *t* tests; ^#^ *p* < 0.05, ^##^ *p* < 0.01 and ^###^ *p* < 0.001 versus vehicle‐treated APP/PS1 mice (Con) by the one‐way ANOVA with post hoc Fisher's LSD, *n* = 6 in each group.

We examined the mitochondrial transmembrane potential (MMP) by FCM analysis. As shown in Figure 5A, the mitochondrial lesions were increased in the AD mice brains than those of age‐matched WT group. The decline in the MMP of the APP/PS1 mice brains was alleviated under LIG‐LPs treatment (Figure 5B). It has been reported that mitochondrial fusion proteins are significantly reduced, whereas the levels of mitochondrial fission proteins, such as Drp1, are increased in the brains of patients with AD.^49^ In the present study, we first compared the expression of fusion and fission proteins between APP/PS1 and age‐matched WT mice brains. As shown in Figure 5C, the levels of mitochondrial fusion protein, MFN2, were significantly lower in the APP/PS1 mice brains. The levels of phosphorylated Drp1 (p‐Drp1) were reduced in the APP/PS1 mice brains. The decreases of an OMM protein, Toom 20, were also observed. We then evaluated the effects of LIG treatment on the above proteins. As shown in Figure 5D, the protein expressions of MFN2 and p‐Drp1 were markedly increased in the LIG‐treated APP/PS1 mice brains compared with those of vehicle‐treated APP/PS1 controls. Under LIG treatment, the levels of Drp1 protein were reduced relative to vehicle‐treated controls in the APP/PS1 mice brains. We then evaluated the mitochondrial morphology in the hippocampus of the APP/PS1 mice by TEM assays. As shown in Figure 5E, the mitochondria were swollen and vacuolated in the hippocampal samples from vehicle‐treated APP/PS1 mice. The double‐layer membrane structure of mitochondria was destroyed. The mitochondrial cristae were obviously broken or lost. The length of mitochondria was reduced, and the number of abnormal mitochondria was increased. However, in the LIG‐LPs‐treated APP/PS1 group, the double‐layer membrane structure of the mitochondria was relatively intact. The cristae of the mitochondria was distinct. The length of mitochondria was increased. Abnormal mitochondria was reduced. Considering the inhibition of mitochondrial fission could prevent neuronal apoptosis,^50^ we further analyzed the levels of anti‐apoptotic and pro‐apoptotic proteins. As shown in Figure 5F and Figure S1, LIG management increased the protein expressions of B‐cell lymphoma‐2 (Bcl‐2), and facilitated the phosphorylation of Bcl‐2 associated death promoter (Bad). The protein levels of a pro‐apoptotic biomarker, Bax, were reduced in the LIG‐treated APP/PS1 mice brains than those of vehicle‐treated APP/PS1 group, suggesting that LIG‐mediated modulation on the mitochondrial fusion and fission might inhibit the apoptosis in the APP/PS1 mice brains.

### 3.6 The neuroprotective effects of LIG on brains of APP/PS1 mice are involved in the regulation of PKA/AKAP1 signaling

**FIGURE 6:**
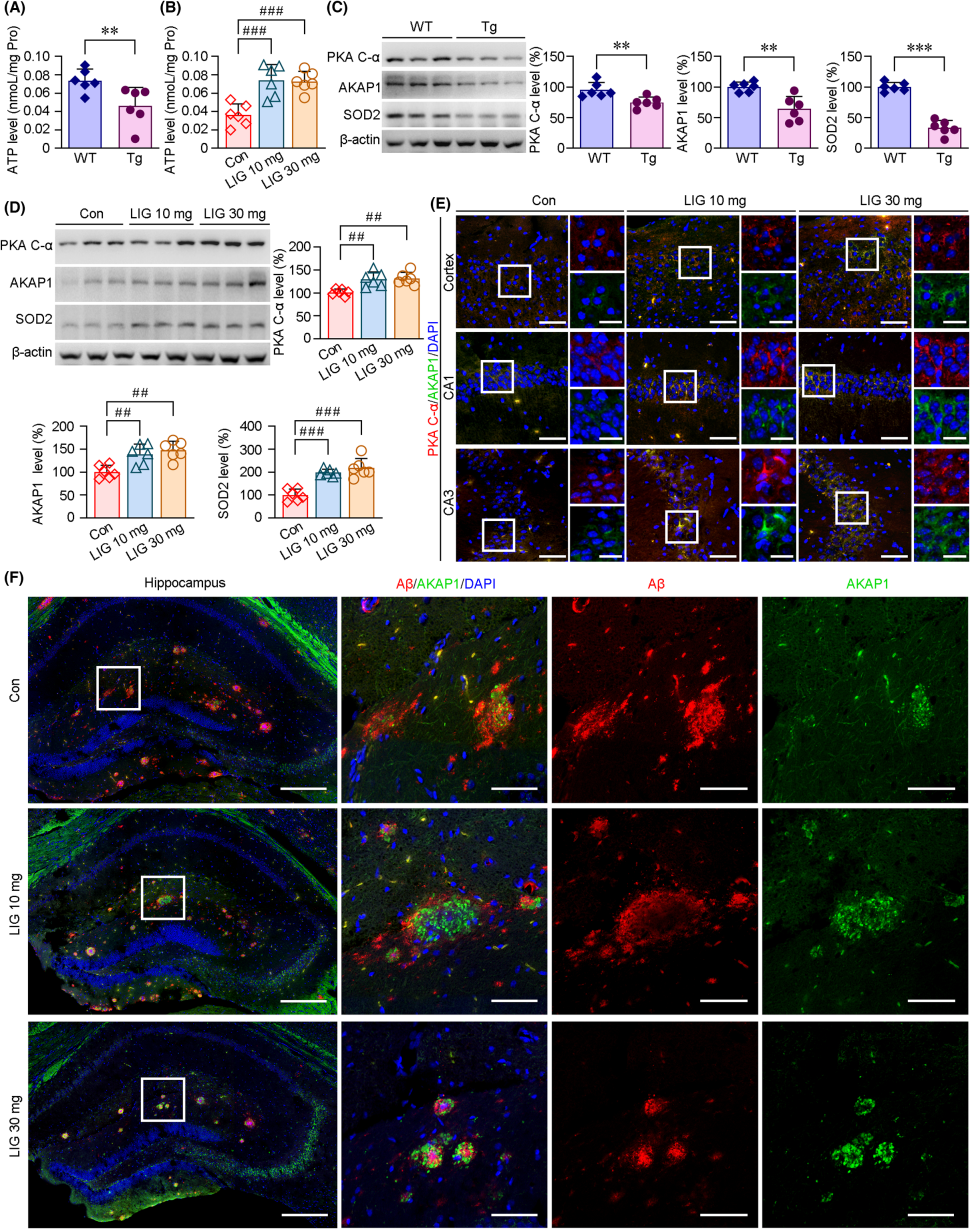
LIG administration upregulates PKA/AKAP1 signaling in the APP/PS1 mice brains. (A) ATP levels in the transgenic (Tg) APP/PS1 and age‐matched wild‐type (WT) C57BL/6 mice brains were assayed. (B) Effects of LIG treatment on the ATP production in the APP/PS1 mice brains. (C) Western blot assays showed the protein levels of PKA, AKAP1 and SOD2 in the WT and Tg mice brains. (D) LIG treatment increased the protein expressions of PKA Cα, AKAP1 and SOD2 compared with those of vehicle controls (Con) in the brains of APP/PS1 mice. (E) Double IF labeling exhibited the protein expressions and distributions of PKA Cα and AKAP1 in the cortical cortex and hippocampal CA1 and CA3 of the APP/PS1 mice. Scale bar = 50 μm. High‐magnification images indicated the immunoreactive staining of PKA Cα and AKAP1. Scale bar = 10 μm. (F) Representative images showing the protein expressions of AKAP1 near the Aβ plaques in the hippocampus of the APP/PS1 mice brains. Scale bar = 200 μm. Large images indicated the regions in the boxes of the left panels. Scale bars = 50 μm. Values represent mean ± SEM, **p < 0.01 and ***p < 0.001 versus WT group by the Student's *t* tests; ^##^ *p* < 0.01 and ^###^ *p* < 0.001 versus vehicle‐treated APP/PS1 mice by one‐way ANOVA with post hoc Fisher's LSD. *n* = 6 in each group.

Mitochondria are the main apparatus for ATP production, and ATP contents reflect the mitochondrial status. We analyzed ATP levels in the brains of our experimental mice. As shown in Figure 6A, ATP contents were significantly lower in the brains of APP/PS1 mice than those of the age‐matched WT group. We then compared the ATP levels between LIG‐ and vehicle‐treated groups in the brains of APP/PS1 mice. Under LIG treatment, the ATP contents were markedly increased in the brains of APP/PS1 mice compared with those of vehicle‐treated APP/PS1 controls (Figure 6B). ATP production is regulated by PKA/AKAP1 signaling.^51^ In the present study, considering the modulatory effects of LIG on mitochondria in the brains of APP/PS1 mice, we investigated whether the LIG‐mediated improvement in mitochondrial fusion and fission is related to the regulation of PKA/AKAP1 signaling. Western blot analysis showed that there were significant decreases in the protein expressions of PKA C‐α and AKAP1 in the brains of APP/PS1 mice compared with those of age‐matched WT group (Figure 6C; Figure S2). The protein levels of SOD2, a downstream target of AKAP1, were also lower in the brains of APP/PS1 mice (Figure 6C; Figure S2). LIG treatment increased the protein expressions of PKA C‐α and AKAP1, and the effects of LIG on regulating the protein expressions of SOD2 and phosphorylation of BAD (p‐Bad) were also observed (Figure 6D; Figure S2). IF staining exhibited LIG‐induced increases in PKA Cα and AKAP1 protein levels (Figure 6E). Interestingly, the distributions of AKAP1 immunoreactive production were increased corresponding to the decreases of Aβ‐plaque deposition in the hippocampus of the APP/PS1 mice (Figure 6F).

**FIGURE 7:**
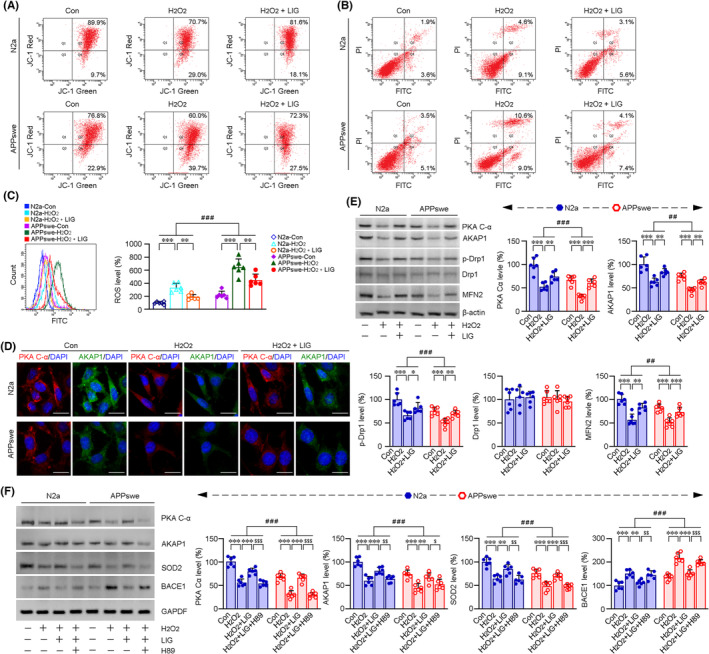
LIG‐mediated protection on mitochondria against oxidative stress is involved in the modulation of PKA/AKAP1 signaling pathway. (A) FCM analysis showed that the decline of MMP triggered by H_2_O_2_ (50 μM, 24 h) was alleviated under LIG administration (pretreatment with 10 μM LIG for 8 h following with the exposure to 50 μM H_2_O_2_ for 24 h) in the N2a cells and N2a cells stably transfected with human β‐amyloid precursor protein Swedish mutation (APPswe). Apoptosis (B) and ROS levels (C) were determined by FCM quantitative assay. (D) IF staining showed the protein expressions and distributions of PKA Cα and AKAP1. Scale bar = 10 μm. (E) Western blot assays showed the alterations of protein levels in the PKA C‐α, AKAP1 and the protein expressions related to mitochondrial fusion and fission under the indicated treatment. (F) The LIG‐mediated regulations on PKA, AKAP1, SOD2 and BACE1 were blocked by H89 (7.5 μM, 4 h). *p < 0.05, **p < 0.01 and ***p < 0.001 versus H_2_O_2_ treatment group; ^##^ *p* < 0.01 and ^###^ *p* < 0.001 versus N2a cells; ^$^ *p* < 0.05, ^$$^ *p* < 0.01 and ^$$$^ *p* < 0.001 versus H_2_O_2_ + LIG treatment group (multivariate ANOVA with post hoc Fisher's LSD). All values are presented in at least three independent experiments.

Interaction of PKA with AKAP1 facilitates the phosphorylation of Drp1, inhibiting the Drp1 and maintaining mitochondrial function.^24^ Interestingly, PKA signaling is an important upstream regulator of BACE1, and Aβ decreases mediated by BACE1 inhibition were PKA‐dependent.^30^ We then assessed the specificity of LIG‐induced increases in the protein expressions of PKA Cα and AKAP1 and investigated whether LIG‐induced modulation on the mitochondrial fusion and fission could provide neuroprotection and inhibition on BACE1 via PKA/AKAP1 signaling pathway. N2a cells or N2a cells stably transfected with human β‐amyloid precursor protein Swedish mutation (APPswe) were subjected to H_2_O_2_ treatment, imitating the oxidative stress status in AD‐related pathology in vitro. FCM analyses showed that LIG treatment mitigated H_2_O_2_‐induced mitochondrial lesions (Figure 7A) and apoptosis (Figure 7B). LIG treatment could ameliorate H_2_O_2_‐triggered increases in ROS (Figure 7C). We then analyzed the protein levels of PKA C‐α and AKAP1 in the N2a and APPswe cells under the indicated treatment. LIG management could reverse H_2_O_2_‐induced downregulation of PKA C‐α and AKAP1 protein expressions (Figure 7D,E), alleviated H_2_O_2_‐primed imbalance of mitochondrial fusion and fission (Figure 7D,E; Figure S3), mitigated H_2_O_2_‐caused decline of SOD2 protein levels (Figure 7F; Figure S3), and lessened H_2_O_2_‐triggered increases of BCAE1 protein expressions (Figure 7F; Figure S3). However, the effects of LIG on PKA C‐α, AKAP1, SOD2 and BACE1 were diminished by an inhibitor of PKA, H89 (Figure 7F; Figure S3).

## 4 DISCUSSION

Oxygen consumption in the brain is the highest in the whole body, whereas the levels of antioxidants are relatively low in the brain. Oxidative imbalance is related to senescence and age‐related disease.^52^ The accumulation of lipid peroxidation is significant in the patients with mild cognitive impairment.^53^ In the AD brain, lipids, proteins and nucleic acids manifest a high level of oxidative modification.^54^ Oxidative stress is a kind of cell damage caused by the imbalance between oxidation and antioxidation, which is closely related to the excessive production of reactive nitrogen species and ROS. Oxidative stress is commonly exhibited in the synapses of the AD postmortem brain^55^ and AD mouse models, and the synaptic deficits appear ahead of Aβ‐senile plaque deposition.^56^ Oxidative stress is one of the early events, and continuous oxidative imbalance influences the magnitude and severity of AD.^57^ Mitochondria are the main organelle of ROS production; accordingly, mitochondria are also the primary attacking target of ROS. The fission and fusion of mitochondria are dynamic processes, which are essential for the maintenance of mitochondrial function. The balance of mitochondrial fission and fusion is crucial for cell survival, free radical homeostasis, Ca^2+^ buffering and mitochondrial quality control.^58^ During mitochondrial fusion, MFN1 and MFN2 facilitate the binding of two adjacent outer membranes of mitochondria; Opa1 mediates the fusion of mitochondrial inner membranes. The accumulation of Drp1 and mitochondrial fission protein 1 in the outer membrane of mitochondria could form helical structures, causing mitochondrial fission.^59^ It has been reported that in AD patients, abnormal mitochondrial metabolism was observed ahead of histopathological changes or clinical manifestations.^60^ Ultrastructural morphometric studies showed that there was significant structural damage of mitochondria in the brain tissues of AD patients: the mitochondrial crista of pyramidal neurons was broken, and the internal structure of the mitochondria from some cases was almost completely lost.^17^, ^61^ The gene levels of *Mfn1*, *Mfn2* and *Opa1* were markedly reduced, whereas the gene levels of *Drp1* and mitochondrial fission protein 1 were elevated in the neurons from the brains of AD patients.^62^ Those above are considered to be important causes for mitochondrial dysfunction and the increases of oxidative stress in AD brains. Additionally, mitochondrial damage‐triggered increases in ROS could promote Aβ pathology.^63^ Importantly, oxidative stress may also be associated with neuroinflammation, neurofibrillary tangles and metabolic disorder of metal ions in the AD brain. Remedying oxidative imbalance is considered a potential strategy for AD treatment. Maintaining the physiological functions of mitochondria may be a critical point for intervention in AD.

Antioxidants, such as polyphenols isolated from brown algae perform the bioactive and neuroprotective effects, reducing ROS before altering other signaling pathways and exhibiting anti‐inflammatory properties.^64^ Isoprenoids, derived from sterols and xanthin, could drive the neuroprotective effects, which is primarily based on their anti‐oxidative radical scavenging.^65^, ^66^ Resveratrol reduces the formation of ROS, downregulates the expression of proteins that can induce oxidative stress and improves the expressions of memory‐related proteins, ameliorating the dyshomeostasis of oxidation–reduction.^67^, ^68^ The properties of antioxidants in the redox regulation of cellular stress response are considered the potential to inhibit, retard or reverse the steps causing neurodegeneration in AD.^69^ Patients with mild or moderate cognitive impairment given an early intervention with antioxidants, colostrinin, exhibited the improvement of their cognitive functions.^70^ The capacity of antioxidants to modify epigenetic markers has increasingly attracted the attention of researchers.^71^ LIG inhibited the production of ROS and increased the expression of antioxidant proteins in a cell model of atherosclerosis.^72^ LIG administration prolonged the length of mitochondria and modulated the expression of mitochondrial fusion and fission proteins in an AD mouse model in vivo.^38^ We propose that the antioxidant properties of LIG may be applicable to the treatment of AD. In the present study, LP‐encapsulated LIG is relatively safe, with a small particle size and good uniformity, which is beneficial to improve the bioavailability of LIG. As exhibited by Golgi staining, LIG treatment alleviated the loss of neurons in the APP/PS1 mice brain. The Aβ‐plaque deposition was reduced in LIG‐treated APP/PS1 mice brains, which might be due to the inhibition of amyloidogenesis and/or Aβ accumulation. The decreases of ROS, MDA and carbonylated proteins indicated LIG‐induced amelioration of oxidative stress in the brains of APP/PS1 mice, and the elevation of GSH/GSSG ratio in the mice brains of the LIG treatment group confirmed the mitigation of oxidative imbalance. Interestingly, compared with the vehicle‐treated APP/PS1 group, the morphology, inner structure and number of mitochondria were amended in the hippocampus of LIG‐treated APP/PS1 mice brains, indicating LIG‐mediated protection of mitochondria. Under LIG administration, the decline in the protein expressions of MFN2 and p‐Drp1 was alleviated, accompanying the decreases of Drp1 protein levels in the brains of APP/PS1 mice, which suggested LIG‐induced improvement of mitochondrial fusion and fission balance. Our results indicated that LIG treatment promoted the quality control of mitochondria in the AD mice brains.

Partially reducing Drp1 might be a potential intervention for AD.^49^ Drp1 could interact with Aβ monomers and oligomers, causing excessive fragmentation of mitochondria. The activation of Drp1 could facilitate the release of cytochrome C from mitochondria, resulting in cell apoptosis.^73^, ^74^ It has been reported that hydrogen peroxide and lipid peroxidation are reduced in the Drp1^+/−^ mice.^49^ Importantly, PKA‐induced phosphorylation of Drp1 inhibits the activity of Drp1, elongating mitochondria through unopposed fusion.^75^ The elevation of cAMP and the expression of PKA catalytic subunit reshape mitochondria into an interconnected network,^76^ and the interaction of AKAP1 with PKA can enhance the phosphorylation of Drp1, inhibiting mitochondrial fission and providing protective effects. In the present study, the decreases in the protein expressions of PKA catalytic subunit and the increases of Drp1 protein levels indicated that mitochondrial dysfunction may be involved in the decline of the PKA signaling in the brains of APP/PS1 mice. LIG treatment increased the protein expressions of PKA and p‐Drp1 compared with those of vehicle‐treated APP/PS1 controls. LIG treatment also induced increases in the protein levels of AKAP1. To investigate the specificity of LIG‐mediated upregulation of PKA/AKAP1 signaling, the effects of LIG were analyzed in H_2_O_2_‐treated APPswe cells in vitro. LIG treatment alleviated H_2_O_2_‐triggered oxidative stress and reduced apoptosis, confirming the neuroprotective effects of LIG that have been previously reported in vitro.^77^ LIG administration reversed H_2_O_2_‐induced increases in Drp1 protein levels, upregulated the protein expressions of AKAP1 and PKA C‐α and affected the AKAP1 downstream target SOD2. However, the above effects were diminished by incubation with an inhibitor of PKA, H89. Zhang and colleagues have reported that the reduction of Aβ is related to the shift of APP processing toward nonamyloidogenic pathway caused by the activation of the PKA.^30^ In our study, we showed that LIG treatment reversed oxidative stress‐induced increases of BACE1 protein levels in vitro, which was consistent with the decreases of Aβ‐plaque deposition in the brains of LIG‐treated APP/PS1 mice in vivo. H89‐casued suppression of LIG on modulating BACE1 confirmed that LIG treatment induced nonamyloidogenic processing of APP through PKA signaling pathway. Our results suggest that LIG‐mediated regulation of mitochondrial function and inhibition of oxidative stress occur via PKA/AKAP1 signaling.

Our study showed that LP packaging facilitated LIG application potential in the treatment of AD. LIG‐LPs improved the learning and memory function of AD mice, reduced Aβ plaque deposition and alleviated apoptosis. We observed LIG‐mediated neuroprotective effects due to the regulation of mitochondrial function homeostasis by upregulating the PKA/AKAP1 signaling.

## AUTHOR CONTRIBUTIONS

Chun‐Yan Wang designed the study. Chun‐Yan Wang, Qi Zhang, Xiangxiang Zhang, Bing Yang, Yan Li, Xue‐Heng Sun, Xiang Li, Ping Sui, Yi‐Bin Wang and Shu‐Yu Tian performed the experiments. Qi Zhang, Xiangxiang Zhang, Bing Yang, Yan Li, Xue‐Heng Sun, Xiang Li, Ping Sui, Yi‐Bin Wang and Shu‐Yu Tian analyzed the data. Chun‐Yan Wang and Qi Zhang wrote the manuscript.

## FUNDING INFORMATION

The work was supported by the National Natural Science Foundation of China (Nos. 81971026 and 81671041), Natural Science Foundation of Jilin Provincial Science & Technology Department in China (No. YDZJ202201ZYTS599) and Natural Science Foundation of Jilin Provincial Educational Department in China (No. JJKH20200455KJ).

## CONFLICT OF INTEREST STATEMENT

The authors declare no conflict of interest.

## Supporting information


Figure S1

Figure S2

Figure S3

Table S1

Table S2

## Data Availability

The data that support the findings of this study are available from the corresponding author upon reasonable request.
