# Supplementary figures and images for: Ligustilide‐loaded liposome ameliorates mitochondrial impairments and improves cognitive function via the PKA/AKAP1 signaling pathway in a mouse model of Alzheimer's disease
Source: CNS Neurosci Ther. 2023 Sep 17;30(3):e14460. doi: 10.1111/cns.14460 (PMC10916432; doi:10.1111/cns.14460)

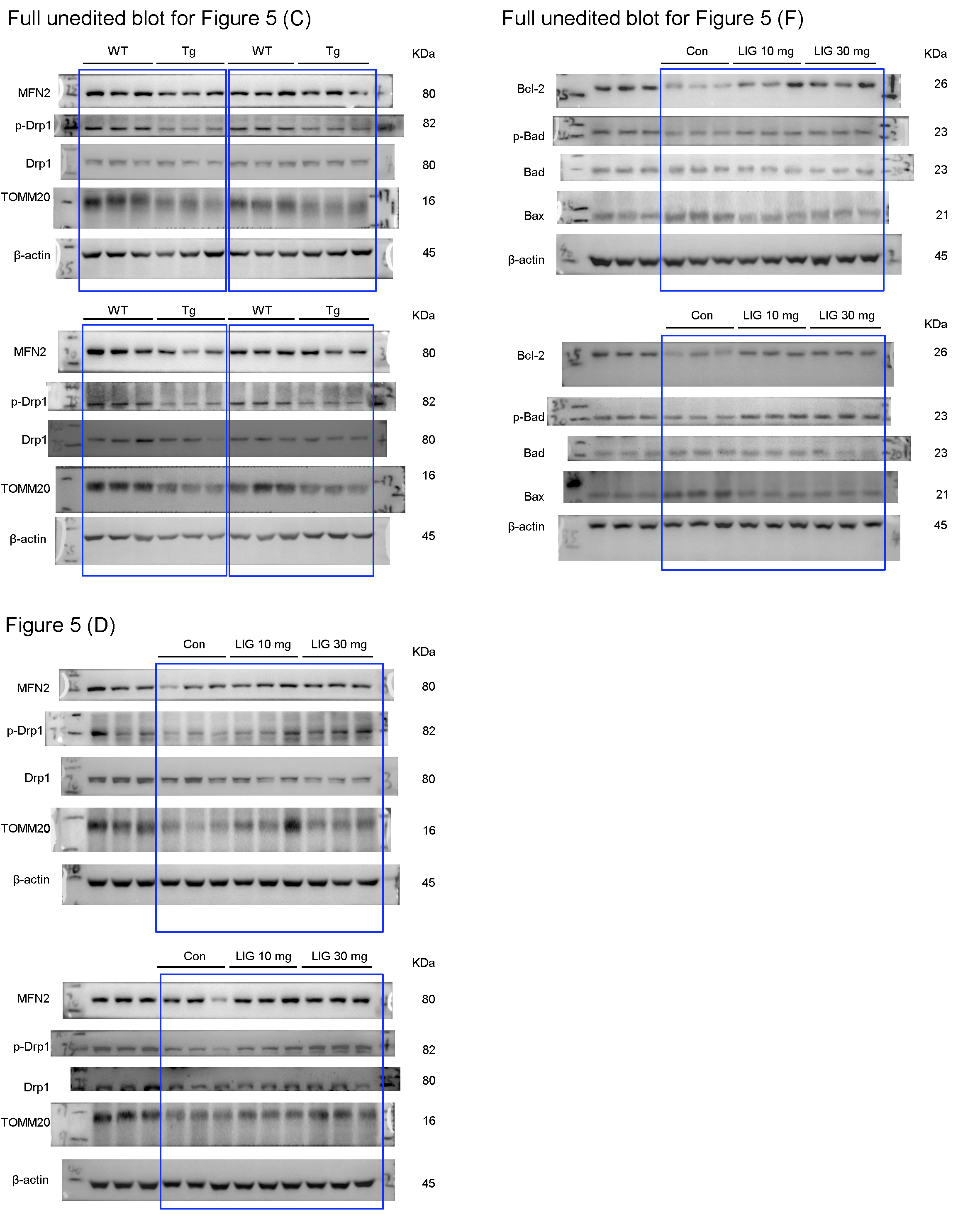

Supplement: Supplementary file 1 — Figure S1 [file CNS-30-e14460-s003.tif]

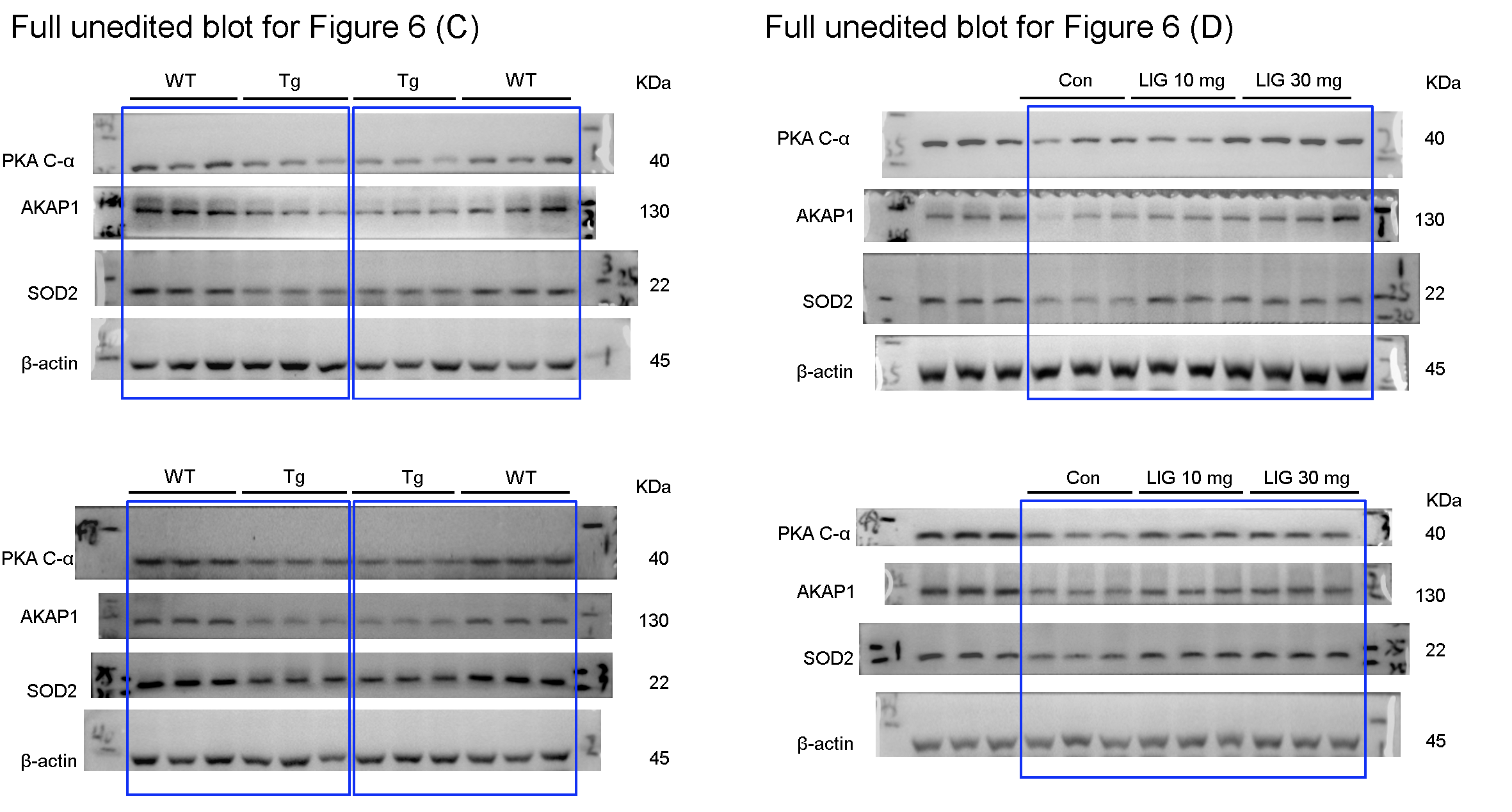

Supplement: Supplementary file 2 — Figure S2 [file CNS-30-e14460-s005.tif]

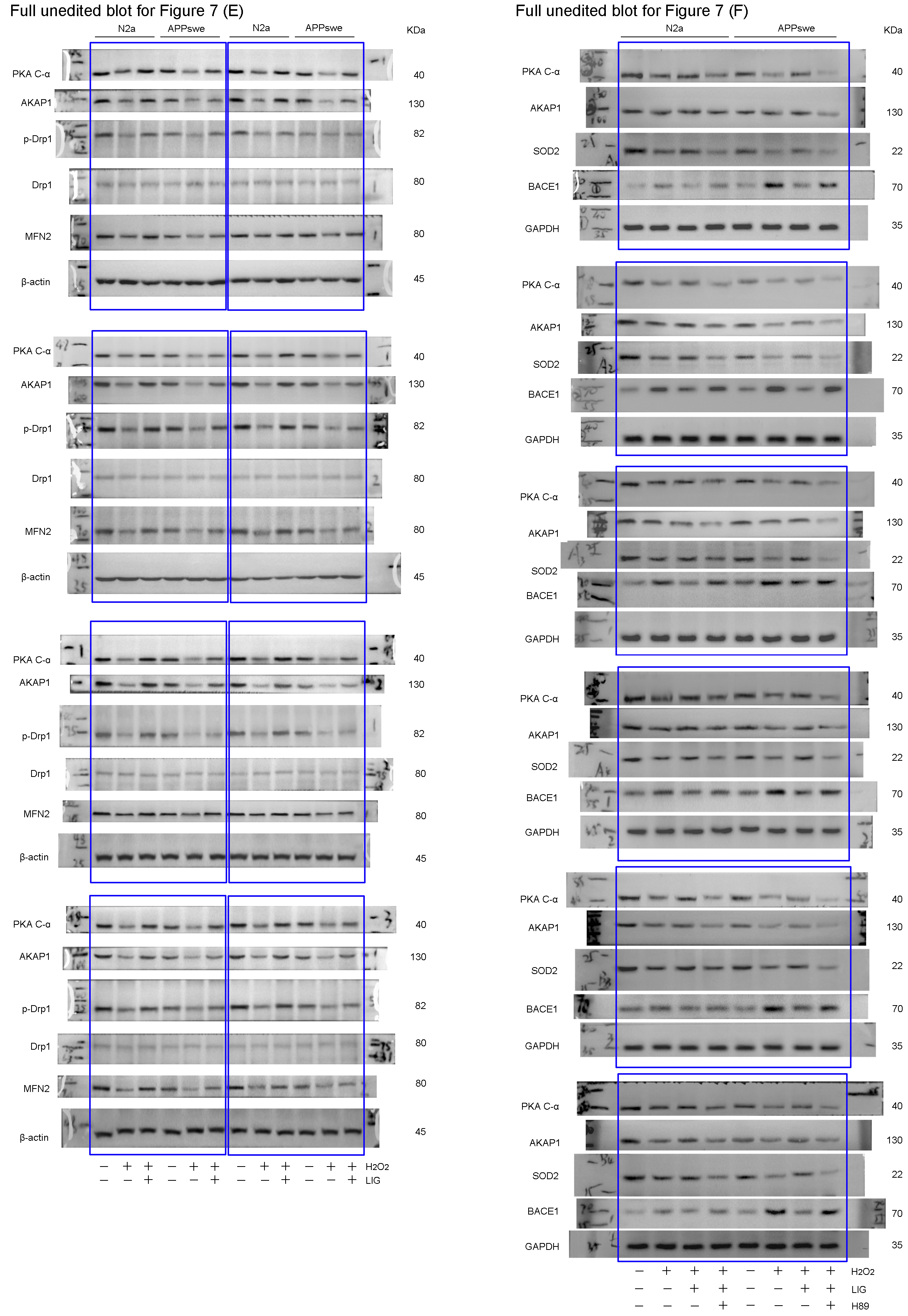

Supplement: Supplementary file 3 — Figure S3 [file CNS-30-e14460-s004.tif]
