# Supplementary material for: Ligustilide‐loaded liposome ameliorates mitochondrial impairments and improves cognitive function via the PKA/AKAP1 signaling pathway in a mouse model of Alzheimer's disease
Source: CNS Neurosci Ther. 2023 Sep 17;30(3):e14460. doi: 10.1111/cns.14460 (PMC10916432; doi:10.1111/cns.14460)
Supplement: Supplementary file 4 — Table S1 [file CNS-30-e14460-s001.docx]

**Supplementary Table S1 KEY RESOURCES**

| **REAGENT or RESOURCE** | **SOURCE** | **IDENTIFIER** |  | |
| --- | --- | --- | --- | --- |
| Antibodies | Supplier | Catalogue number | | Molecular Weight (kDa) |
| Mouse anti-Aβ | Sigma-Aldrich | Cat# A5213 | | 5 |
| Mouse anti-Drp1 | Santa Cruz Biotechnology | sc-271583 | | 80 |
| Mouse anti-PKA C-α | Santa Cruz Biotechnology | sc-28315 | | 40 |
| Mouse anti-TOMM20 | Abcam | ab56783 | | 16 |
| Rabbit anti-Aβ | Cell Signaling Technology | Cat# 8243S | | 5 |
| Rabbit anti-AKAP1 | Cell Signaling Technology | Cat# 5203 | | 130 |
| Rabbit anti-BACE1 | Cell Signaling Technology | Cat# 5606 | | 70 |
| Rabbit anti-Bcl-2 | Cell Signaling Technology | Cat# 3498 | | 26 |
| Rabbit anti-Bad | Cell Signaling Technology | Cat# 9268 | | 23 |
| Rabbit anti-β-actin (D6A8) | Cell Signaling Technology | Cat# 8457 | | 45 |
| Rabbit anti-Bax | Abcam | ab182733 | | 21 |
| Rabbit anti-DRP1 (phospho S637) | Abcam | ab193216 | | 82 |
| Mouse anti-GAPDH | Aksomics | KC-5G5 | | 35 |
| Rabbit anti-MFN-2 | Cell Signaling Technology | Cat# 9482 | | 80 |
| Rabbit anti-8-OHG | Santa Cruz Biotechnology | sc-393871 | | - |
| Rabbit anti-Phospho-Bad (Ser155) | Cell Signaling Technology | Cat#9297 | | 23 |
| Rabbit anti-SOD2 | Cell Signaling Technology | Cat# 13141 | | 22 |
| Rabbit anti-TOMM20 | Cell Signaling Technology | Cat# 42406 | | 16 |
| Critical Commercial Assays | Supplier | Catalogue number | |  |
| Annexin V-FITC Apoptosis Detection Kit | Cell Signaling Technology | Cat# 6592 | |  |
| Enhanced ATP Assay Kit | Beyotime Biotechnology | S0027 | |  |
| FD Rapid GolgiStainTM Kit | FD Neurotechnologies, Inc., USA | PK401 | |  |
| MDA Detection Kit | Nanjing Jiancheng Bioengineering Institute | A003-1-2 | |  |
| MitoProbe™ JC-1 Assay Kit | Thermo Scientific | Cat# M34152 | |  |
| Toal glutathione/Oxidized glutathione assay kit | Beyotime Institute of biotechnology | A061-2-1 | |  |
