# Supplementary material for: Ligustilide‐loaded liposome ameliorates mitochondrial impairments and improves cognitive function via the PKA/AKAP1 signaling pathway in a mouse model of Alzheimer's disease
Source: CNS Neurosci Ther. 2023 Sep 17;30(3):e14460. doi: 10.1111/cns.14460 (PMC10916432; doi:10.1111/cns.14460)
Supplement: Supplementary file 5 — Table S2 [file CNS-30-e14460-s002.docx]

**Supplementary Table S2**. The characteristics of the LIG-LPs and LPs

| Sample | Intensity size (nm) | Zeta potential (mV) | PDI |
| --- | --- | --- | --- |
| LPs | 115.09 ± 8.27 | -28.11 ± 8.43 | 0.115 ± 0.06 |
| LIG-LPs | 85.29 ± 7.53 | -23.68 ± 5.77 | 0.147 ± 0.03 |
